# Wet shells and dry tales: the evolutionary ‘Just-So’ stories behind the structure–function of biominerals

**DOI:** 10.1098/rsif.2022.0336

**Published:** 2022-06-15

**Authors:** Robert Lemanis, Kian Tadayon, Elke Reich, Gargi Joshi, Richard Johannes Best, Kevin Stevens, Igor Zlotnikov

**Affiliations:** ^1^ B CUBE – Center for Molecular Bioengineering, Technische Universität Dresden, Dresden, Germany; ^2^ Institut für Geologie, Mineralogie und Geophysik, Ruhr-Universität Bochum, Bochum, Germany

**Keywords:** structure–function, biominerals, mechanical properties, adaptation

## Abstract

The ability of evolution to shape organic form involves the interactions of multiple systems of constraints, including fabrication, phylogeny and function. The tendency to place function above everything else has characterized some of the historical biological literature as a series of ‘Just-So’ stories that provided untested explanations for individual features of an organism. A similar tendency occurs in biomaterials research, where features for which a mechanical function can be postulated are treated as an adaptation. Moreover, functional adaptation of an entire structure is often discussed based on the local characterization of specimens kept in conditions that are far from those in which they evolved. In this work, environmental- and frequency-dependent mechanical characterization of the shells of two cephalopods, *Nautilus pompilius* and *Argonauta argo*, is used to demonstrate the importance of multi-scale environmentally controlled characterization of biogenic materials. We uncover two mechanistically independent strategies to achieve deformable, stiff, strong and tough highly mineralized structures. These results are then used to critique interpretations of adaptation in the literature. By integrating the hierarchical nature of biological structures and the environment in which they exist, biomaterials testing can be a powerful tool for generating functional hypotheses that should be informed by how these structures are fabricated and their evolutionary history.

## Significance statement

The impressive material properties of biomineralized tissues have motivated a wealth of research into the characterization of their macro-, meso- and nano-scale features. Traditionally, an isolated feature set at one scale is investigated under dehydrated conditions. These results are then combined with some postulated function to frame these features as adaptations of the animal. We demonstrate that the properties at one scale cannot always be predicted using the properties from a different scale and the necessity of testing biological tissues in environments similar to their natural state. To understand the origins of these features one needs to consider not just the potential functions but also the growth of the structure and the phylogeny and ecology of the organism.

## 1. Introduction

For over 500 million years, starting—perhaps—as early as the Cryogenian [1], a planet-wide set of iterative experiments have been running, modifying the composition and morphology of biomineralized hard parts of living organisms into a diverse array of structures: from spicules, plates and sclerites, to teeth, shells and skeletons. These structures share a similar organization in the sense of integrating a mineral component in an organic matrix, with more derived architectures exhibiting a hierarchical system of morphology [2,3]. From a functional perspective, the combination of a compliant phase and a stiff phase, coupled with the hierarchical nature of these structures, contributes to a variety of stiff, tough and stable end-products with the capability to deform, creep, recover, undergo stress relaxation, absorb energy, filter frequencies and more. These are often well beyond what would be expected based on a simple homogeneous mixture of the constituent parts [4]. Unsurprisingly, the structure–function relationship of these tissues comprises a significant fraction of the literature on biomaterials, with a focus on detailing the nano-, micro- and macro-scale features that could contribute towards the development of these impressive properties. A common trend easily observable in much of this literature is the effort to paint all such features, necessarily, as outcomes of adaptive evolution—with adaptation here referring both to the process by which an organism becomes fitted to its environment and to the outcomes of this process.

Through much of the 1900s, rooted in traditions such as the idea of the *Allmacht* of natural selection [5], there was a tendency among biologists to view animals as atomized parts and search for adaptive explanations of these parts divorced from either a view of the organism as a cohesive whole, or any constraints on the outcomes of the evolutionary process [6,7]. Although the goal of biomaterials research is not necessarily to probe evolution, as stated previously, the way evolution is evoked in the research often echoes the trends of this early, atomized view of organisms. Are local gradients in crystal aspect ratio [8], mineral bridges between crystals [9] and screw-like dislocations [10,11] adaptations to increase strength and toughness or are they necessarily formed during the self-assembly of the mineral [12–14]? Of course, these two aspects are not mutually exclusive; the function of a part and how that part forms are two different topics. Although there is no *a priori* reason to invoke functionality if fabrication presents a sufficient explanation, especially when functionality is used as ‘window dressing’ rather than the topic being explored. Even more so when the functionality of the feature in question is viewed without accounting for rest of the organism, assuming that the mechanical effect of these small-scale features simply scales up to the organismal level and overlooking modularity [15]—the potential interactions between different components of the organism both in growth and in function. This assumption is even more glaring in cases where the environment of the biomaterial during life is ignored when measuring its material properties; biomaterials are often tested in dehydrated states under simple quasi-static loading conditions.

Consider, for example, one of the most studied biomaterials: nacre. Nacre has long been thought remarkable for its strength and toughness relative to pure aragonite largely due to the interplay of several different features including: nanoasperities [16,17], dove tailing [18] and tablet interlocking [19]. Yet most of the experiments on nacre of molluscan shells have been performed on dehydrated samples*,* with rare exceptions [20,21], and despite the knowledge that moisture content has a significant effect on measured material properties [22–24]. Furthermore, the mechanical ‘superiority’ of nacre is commonly discussed while ignoring that it is only one part and, in many cases, only a small part of the entire shell [25,26].

The goal of this contribution is to examine the adaptationist narrative as it manifests in the field of biomaterials and suggests some ways such narratives can be framed in a way that does not present evolution as a simple optimization process. Largely drawing on broader evolutionary frameworks, such as constructional morphology [7,27,28], that integrate multiple factors that shape organismal form, such as the environment, phylogeny, fabrication and function. In the light of the stated goal, much of the discussion here will focus on function as it is the aspect of our broader framework that biomaterials research can directly address. As we can see in the examples from the previous paragraph, this adaptationist narrative commonly manifests from discussions resulting from measuring material properties and then relating those properties to some adaptive scenario. How these properties are measured, the scale at which the measurements are done, and the connection to functional morphology are, therefore, all of primary concern. The questions then are as follows: How do moisture and scale affect the results of some common tests? How do they scale up to reflect the performance of an entire biomaterial? Can these be put into the perspective of adaptive evolution? To explore this, we compared environmentally dependent static and dynamic properties of two different but related biogenic mineralized structures at different length scales.

## 2. Results

**Figure 1.**
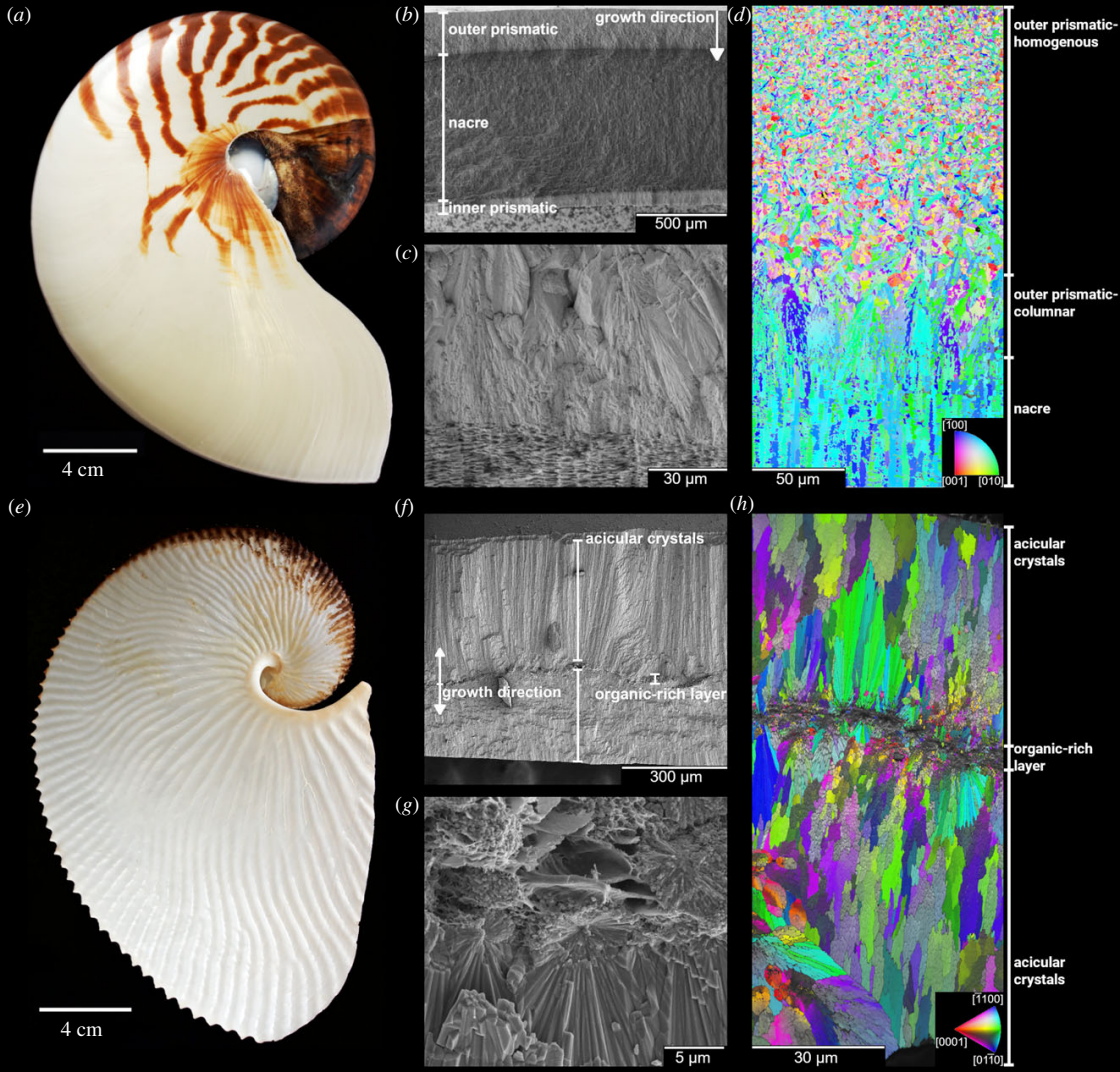
Overview of the two animal shells used in this study: the aragonitic shell of *Nautilus pompilius* (*a*–*d*) and the calcitic shell of *Argonauta argo* (*e*–*h*). The shell of *N. pompilius* (*a*) is composed of three primary layers (*b*): the outer prismatic layer that transitions into the nacre layer (*c*), which then transitions into the inner prismatic layer. EBSD of *N. pompilius* shows a clear increase in texture going from the homogeneous zone (top of the image) down to the columnar and finally the nacreous zones (*d*). The shell of *A. argo* (*e*), in contrast with *N. pompilius*, grows bi-directionally from a central organic layer (*f*). Most of the thickness of the shell is formed by acicular calcite crystals that grow in conical clusters. These clusters begin as spherulites in the organic layer (*g*). The conical crystal clusters that make up the shell of *A. argo* are visible within the EBSD map and show a co-orientation within the clusters, the blue/green clusters near the image centre (*h*). Much of the variation in orientation seen in the image is due to neighbouring clusters going into and out of the plane. The colour-coded inverse pole figures have their reference direction normal to the image plane.

The shells of molluscs present an excellent medium to test these material properties due to several decades of research into their structure and mechanics [20,29–31]. The aforementioned nacre ultrastructure is taken from the external shell of the cephalopod *Nautilus pompilius* (figure 1*a*–*d*). The nautilid shell is predominately nacre sandwiched between two thin prismatic layers [32,33]. This shell grows uni-directionally in thickness, starting at the homogeneous zone of the outer prismatic layer [13,34] and progressing to the columnar zone, which then transforms into nacre (figure 1*c*). The columnar nacre layer eventually transforms into the inner prismatic layer. This architecture is standard for externally shelled cephalopods as fossil ammonoids, nautiloids and basal coleoids also share this three-layered shell structure [33,35,36]. A notable exception to this, and the second structure studied for this work, is the shells of the pelagic octopus genus *Argonauta* (figure 1*e*–*h*).

Well described by Aristotle, these pelagic octopuses were once thought to have been parasitic in the sense that they would steal the shell of some other *Carinaria*-like animal to live in [37]. It was not until the pioneering work of Villepreux-Power, who was able to raise argonauts in aquariums, that the ability of female argonauts to construct the shell themselves was discovered [38]. Unlike other mollusc shells that are formed by the mantle, this shell is formed through two membranes on the dorsal arm pair in female argonauts [38]. This observation emphasizes the fact that the shell is non-homologous to the shells of other molluscs. This purely calcitic shell is derived through a different developmental pathway [38] and possesses different shell matrix proteins compared to other cephalopod shells while also lacking chitin in the organic shell matrix [39]. Unlike the uni-directional construction exhibited by the nautilid shell, the argonaut constructs its shell bi-directionally in thickness from a central organic layer (figure 1*f*). The long, thin crystals that form the bulk of the shell thickness begin as spherulites within the organics-rich layer (figure 1*g*) that grow outwards in conical clusters. Though the ultrastructure, crystallography and geochemistry of the shell have been previously investigated [40–46], the mechanics of the shell are, to our knowledge, unexplored beyond notes of the shell being fairly flexible when wet [38]. The unique construction of the argonaut shell, with a large central organics-rich layer, and the relative simplicity of the organization of the mineral structure compared to *Nautilus* make for an interesting comparison between the two architectures. Previous work done by the authors has shown a minimal effect of humidity on indentation properties of *N. pompilius*, which further provides an opportunity to compare the effects of moisture and if the shell of *A. argo* indeed becomes flexible when wet.

### 2.1. Shell structure and texture

The different architecture of the two shells arises from fundamentally different growth modes discussed previously. These different growth modes also explain the differences in crystallographic texture seen in the electron backscatter diffraction (EBSD) images (figure 1*d*,*h*). EBSD results taken from *A. argo* agree with previous work [44]. The coherence appears lower at the immediate edges of the central organic zone while the fully developed acicular crystals appear co-oriented (figure 1*h*). Much of the variation in crystal orientation seen in the shell wall is due to neighbouring fibre clusters growing into/out of the image plane. Unlike in *N. pompilius*, where the organics are largely dispersed throughout the shell thickness between the mineral units, *A. argo* shows a concentration of organics in the centre of the shell (figure 1*g*,*h*).

Thermogravimetric analysis (TGA) was performed to test whether this central organic layer contributed to an overall higher weight per cent of the organic phase in the argonaut shell. TGA results show slightly different levels of organics between the two samples; *A. argo* has a higher relative organic content: 7.4% compared to *N. pompilius* at 6.1%. In order to test whether the differences between *N. pompilius* and *A. argo* described here lead to differences in their mechanical performance, both shells were subject to a series of quasi-static and dynamic mechanical tests at multiple length scales to characterize potential variations in trends and sensitivities to moisture.

### 2.2. Nano-scale analysis

**Figure 2.**
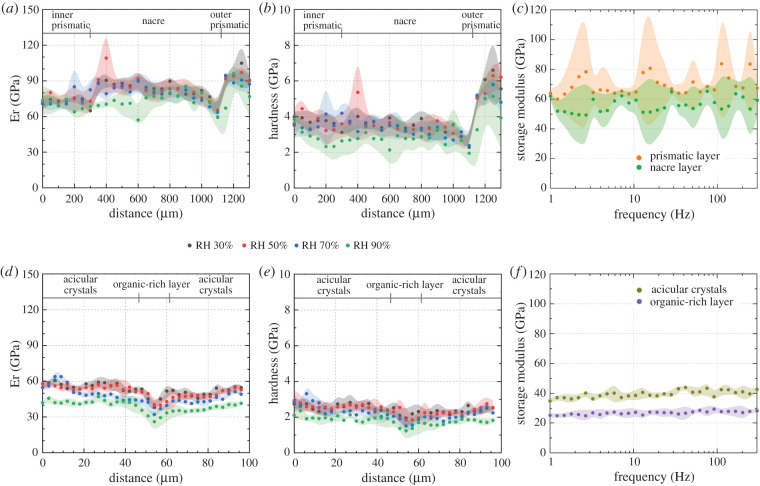
Mechanical characterization on the nano-scale. Reduced modulus (Er) and indentation hardness graphs were calculated from two indentation maps from *Nautilus pompilius* (*a*,*b*), covering an area of 1300 × 200 µm^2^ and *Argonauta argo* (*d*,*e*) covering an area of 96 × 36 µm^2^. The mean values presented in the graphs were made by averaging indentation results across a row of indents made at the same height. Shaded regions represent ±1 s.d. of the averaged data. NanoDMA experiments performed on a cross-section of the shell of *N. pompilius* (*c*) and *A. argo* (*f*) at a relative humidity of 90%.

Quasi-static nanoindentation line-scan tests were performed on the entire cross-sections of *N. pompilius* (figure 2*a*,*b*) and *A. argo* (figure 2*d*,*e*) at a range of relative humidities, from 30% to 90%. The obtained results show a general decrease of reduced modulus and hardness with increasing moisture content, though the effect is structure dependent in *N. pompilius* compared to *A. argo*. *A. argo* shows a higher sensitivity to water content with a greater change in reduced modulus and hardness at higher humidities compared to *N. pompilius*. For example, the global average reduced modulus decreases by 10% in *N. pompilius* and 26% in *A. argo* when comparing the results at 30% RH with 90% RH.

Nano-dynamic mechanical analysis (nanoDMA) was then employed to characterize potential differences in local dynamic properties at high humidity. Dynamic nanoindentation tests performed on a cross-section of *N. pompilius* (figure 2*c*) and *A. argo* (figure 2*f*) at 90% RH show a similar variation in stiffness in major ultrastructural parts of the shell. Like in the quasi-static tests, the organics-rich layer in *A. argo* shows lower modulus values compared to the mineral phase while the prismatic layer shows higher maximal values compared to nacre in *N. pompilius*. An interesting, and unexpected, observation is the emergence of a slight frequency-dependent response mostly visible in the argonaut mineral phase (figure 2*f*) but not present in the nautilid shell.

### 2.3. Macro-scale analysis

**Figure 3.**
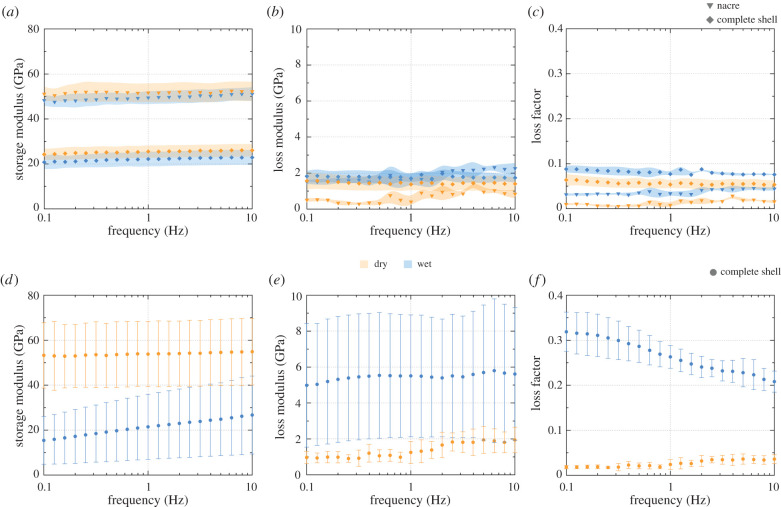
Mechanical characterization on the macro-scale. Storage modulus, loss modulus and loss factor versus frequency graphs for *Nautilus pompilius* (*a–c*) and *Argonauta argo* (*d–f*), respectively. Inverted triangles, diamonds and circles represent data obtained from *N. pompilius* nacre only, complete *N. pompilius* shell and complete *A. argo* shell, respectively. For the data of *N. pompilius*, the shaded regions represent ±1 s.d. of the averaged data. The error bars for the *A. argo* plots, while also calculated as ±1 s.d., show a greater spread compared to *N. pompilius* due to the geometric variation of the beams cut from the shell. In this regard, most of the ‘error’ for *A. argo* is due to geometric differences between the two sides of the same beam that were averaged together for each point.

Classical DMA experiments were performed to see how this frequency dependence scales up to the macro-scale and how it is affected by the internal architecture and the morphology of the shell. Rectangular samples were cut from the edge of the aperture from both *N. pompilius* (figure 3*a*–*c*) and *A. argo* (figure 3*d*–*f*) for dynamic three-point bending tests under ambient ‘dry’ conditions and while fully immersed in water. In the case of the nautilid, two types of samples were tested: an intact shell and segments where the prismatic layers were gently polished away. Furthermore, due to the wavy morphology of the argonaut shell (figure 1*e*), the samples were bent on both sides. This geometric irregularity resulted in the high spread of the results (figure 3*d*–*f*). It is important to mention that calcium carbonate, both calcite and aragonite, are mechanically anisotropic materials [47,48] and, therefore, the direction of load application has a significant effect on the obtained mechanical results on all scales. Hence, in this work, indentation experiments were performed on shell cross-sections, which is also the direction of stress generation during macroscopic bending experiments during macro-scale analysis.

The most striking outcome of this set of experiments is that the storage modulus of pure nautilid nacre is two- to three-fold larger than that of the complete shell, having the outer prismatic architectures intact (figure 3*a*). Similar to the nano-scale, all specimens show a decrease in storage modulus (figure 3*a*,*d*) and an increase in loss modulus (figure 3*b*,*e*) under wet conditions. Comparing the change in storage modulus between ‘dry’ and wet conditions averaged across all frequencies: *N. pompilius* nacre shows an average decrease of 4.5%, the complete *N. pompilius* shell shows an average decrease of 13.0%, while the argonaut samples show an average decrease of 60.6%. Similar averaged comparisons of loss factor (tan delta) also show the separation of these three groups (figure 3*c*,*f*). The loss factor increases under wet conditions. The argonaut shell shows a very large increase in loss factor when wet, with an average increase of 922.0%. *N. pompilius* nacre shows a higher increase of 180.8% compared to the complete shell with an increase of 43.6%. Nevertheless, the absolute value of loss factor is significantly higher in wet argonaut than in any other structure, wet or dry.

The frequency-dependent response previously seen in nanoDMA for *A. argo* is recreated at this length scale but with a much more pronounced effect despite being measured at much smaller frequencies range. In *N. pompilius*, both nacre and the complete shell also show a slight frequency-dependent response on the macro-scale though not as noticeable compared to *A. argo*. While both shells show a general stiffening with increasing frequency and a higher dependence on strain rate while immersed, the observed effect is greater in the argonaut shell compared to any nautilid sample.

## 3. Discussion

Decades of research has undoubtedly advanced our knowledge on morphogenesis, structure and mechanical performance of biological materials. Nacre is an excellent example of how furthering our understanding of fine-scale morphology can also advance our understanding of material properties [16,18,49,50]. This greater understanding has been used to create increasingly more sophisticated and robust biomimetic structures by incorporating features, such as asperities and mineral bridges [51,52]. Indeed, this is a major goal of this field of research. However, the common invocation of functional adaptation leads to an inevitable question: how has research in this vein improved our understanding of nacre evolution and biomineralization, or even of any biomaterial?

While nacre is clearly well characterized, the argonaut shell represents a significant unknown in this study. The ultrastructure and crystallography are known [41,43,53], but its mechanics are poorly understood. The argonaut shell also does not share the more common molluscan structural motifs, such as nacre, crossed-lamellar or prisms; nor does it grow in a similar manner. The spherulitic-fibrous structure of the argonaut shell exhibits notably different mechanical behaviour compared to the familiar spherulitic-prismatic/nacreous shell, specifically in response to high humidity and water immersion. While on the nano-scale the *N. pompilius* shell shows some reduction of reduced modulus in certain regions (figure 2*a*), *A. argo* shows a systematic reduction as a function of relative humidity (figure 2*d*). At the macro-scale, this difference is significantly enhanced (figure 3*a*,*d*). The more extreme dependence in the argonaut shell suggests either a greater water absorption capacity or perhaps some different interaction with water owing to a different organic matrix compared to the nautilid shell [39]. However, if one had compared the properties of these shell structures in their ‘dry’ state [8,54–57], almost no difference in their mechanical performance would have been reported. It should be noted, however, that the rehydrated state does not necessarily capture the original properties of the shell in life due to the potential impact of soft-tissue degradation on the mechanics of the tissues [58,59], though the actual potential impact of this degradation is unknown.

An even more intriguing difference between the two shells is revealed by a comparison at different frequencies. The emergence of a strong frequency-dependent response in the argonaut shell during macroscopic DMA (figure 3), despite the lack of a significant response in nanoDMA (figure 2), indicates some additional mechanism not present at the nano-scale. Also here, if one were to report frequency-dependent mechanical properties of the argonaut based on nano-scale measurements only—a common practice in biomaterials study—a vital aspect of the mechanical performance of this shell would have been overlooked. Surprisingly, combining two ultrastructures that show almost no frequency-dependent behaviour on the nano-scale (figure 2*f*) results in the formation of a material with a pronounced dependency on load application rate (figure 3*d*,*f*). In the nautilid shell, however, frequency-related effects are slight enough to be potentially overlooked on any scale.

We cannot determine the exact mechanisms behind the observed mechanical behaviours; however, we can speculate about their possible dynamics. Both shells have a similar organic content, the mechanical properties of which are assumed to be strongly dependent on moisture acting as a plasticizer. It is expected to contribute to an increased deformability and viscoelasticity of these biomineralized structures [24]. Nevertheless, the nature of the ultrastructures and the arrangement of the constituent materials in space are key to their performance. While nacre has a number of features that limit inter-tablet movement, such as nanoasperities, mineral bridges and dove tailing [60,61], these reinforcing mechanisms were not observed in the prismatic ultrastructure nor the argonaut ultrastructure. Furthermore, the organic phase in the nautilid shell is largely spread throughout thin lamellae in nacre, whereas in the argonaut shell, it is concentrated in relatively large volumes, both in between crystal units [45] and at the centre of the shell (figure 1*g*). These characteristics likely permit a relatively large deformability of the argonaut shell at the macro-scale compared to nacre that has a fairly limited range of motion regardless of the properties of the organic phase. In both cases, these effects cannot possibly be registered by nanoindentation where mostly the mineral phase is probed.

Indentation of hydrated nacreous and prismatic ultrastructures in *N. pompilius* yields reduced modulus values of around 70 GPa and upwards of 80 GPa, respectively (figure 2*a*). Macroscopic DMA measurements are performed in three-point bending mode, meaning that the largest stresses develop along the outer edges of the sample. In nacre specimens, these stresses would be borne by the nacre tablets, with all of their reinforcing mechanisms that limit deformation. However, in the complete shell samples, these stresses are borne by the prismatic layers (figure 1*b*) that, similarly to the shell of the argonaut, lack these reinforcing mechanisms. Therefore, bending of the complete shell shows a storage modulus in the range of 20–25 GPa despite most of the bending stresses occurring within the prismatic layer, which has a higher storage and reduced modulus compared to nacre when measured on the nano-scale (figure 3*a*). Bending of pure nacre shows a significantly higher storage modulus of approx. 50 GPa (figure 3*a*).

When summarizing the experimental mechanical data, the two shells, having very similar elastic modulus values of approximately 20 GPa in their hydrated state demonstrate very different scale-, humidity- and frequency-dependent characteristics. The shell of *N. pompilius* appears to be a ‘perfect’ architecture that combines stiffness, toughness and strength provided by the inner nacreous ultrastructure with deformability provided by the outer prismatic ultrastructures. The shell of *A. argo* seems to dissipate mechanical energy through its viscoelastic response and therefore is extremely sensitive to relative humidity and load application rate, whereas the properties of *N. pompilius* are only slightly sensitive to both. We can also argue, following the tradition discussed in the Introduction, that this study provides a paradigm example of functional adaptation where two externally shelled cephalopods evolved to provide the organisms with mechanical stability and protection using two very different but thoroughly ‘designed’ strategies. However, this leads back to the question posed at the beginning of the discussion: how or even if the obtained data really improved our understanding of the evolution of these shells.

To address this question, we will draw on two examples from the literature to show how the invocation of evolution and the assumption of adaptation have shaped this discussion. The first example deals with the shell of the familiar *Nautilus*. In this study, samples taken from the *Nautilus* sp. shell were indented and subjected to three-point bending experiments to measure bending strength, study crack propagation and calculate the fracture toughness for the organic/mineral components. The authors argue that the *Nautilus* sp. shell ‘exhibits an outstanding environment adaptability in the deep sea’ [55]. However, there are two points to make with regard to the conclusions of this paper. The first is that it is unclear how representative the reported values are of the actual shell during life since all of the experiments were performed on dehydrated specimens. The second concerns the assertion built upon these data: that the spherulitic-prismatic/nacreous shell is an adaptation of extant nautilids to the deep sea.

In a critique of the panselectionist argument that natural selection is wholly sufficient to explain form, Gould & Lewontin [6] invoke the ‘Just-So’ stories of Rudyard Kipling to describe the manifested narrative of these arguments. These stories are framed such that adaptation to some aspect of the current ecology is the ultimate cause of the organism's phenotype. In the case of *Nautilus*, the prismatic/nacre architecture is common in nautiloids older than the genus *Nautilus* [62–64] as well as other externally and internally shelled cephalopods [36]; it seems more likely that the species of *Nautilus* inherited this ultrastructure from a nacre bearing ancestor. While this ultrastructure may not be an adaptation formed by *Nautilus* specifically, we can ask: is there is a connection between a deep-water habitat and the spherulitic-prismatic/nacreous architecture? However, the fossil record argues against a possible connection. The habitats of nautiloids and ammonoids span from shallow, coastal environments to open ocean and deeper water environments without any variation in the basic shell ultrastructure [65–69]. How can the spherulitic-prismatic/nacreous shell be an adaptation to deep water if it is also commonly present in shallow-water cephalopods as well? We can continue to ask questions in this vein, for example, does this shell architecture originate in a deep sea group or could the utility of the shell in the deep sea be a case of exaptation? We can also ask more targeted questions related to the results of the mechanical tests: does the high fracture toughness or bending strength improve the shell resistance relative to some other possible ultrastructure if we consider the actual strain caused by water pressure or the bite force of a known predator? The point of these questions is not to answer them here but to illustrate the kind of queries that should be asked when trying to meaningfully discuss the potential adaptational value of these structures. Simply because improved performance, such as higher bending strength or toughness, of a structure is shown compared to other ultrastructures does not automatically mean that this is the function of the structure or even that the structure is an adaptation at all. To address these questions one has to have an understanding of the ecology of the animal, i.e. the forces the organism is subjected to, as well as its evolutionary history. Finally, the origin of morphology extends beyond functional constraints and also has to include other factors, such as fabrication, phylogeny and environment [7]. This type of approach can be applied to our second example from the literature.

An interesting observation to be made about biomineralized structures across all clades is that their smallest building blocks tend to be on the nanometre-sized scale [70,71]. The potential importance of this was modelled under tension by treating the organic components embedded within mineral units as flaws and calculating the fracture strength of the ‘flawed’ crystal [70]. By calculating the fracture strength at different sizes of the mineral units, the authors concluded that there is a critical length at which the mineral building blocks become insensitive to flaws and their fracture strength is near that of a perfect crystal. This impressive result is then used to suggest that the basic nano-scale theme of biomineralized structures is driven by adaptation towards maximizing fracture strength and flaw tolerance [70]. The authors note that there are other constraints at play, such as the volume fraction of the components, molecular size and other biochemical factors. However, we would like to expand this discussion of constraints by considering the same types of questions we did with the previous example.

The assumption that the nano-granular texture of biominerals is driven by adaptation implies the existence of non-nano-granular textures in early biominerals that are then, due to some external forces, driven by functional demands to nano-granularity. This begs the question: what kind of texture did early biomineralizers possess? This question is not trivial due to the incomplete nature of the fossil record and diagenesis of early shells. That being said, some of the earliest biomineralized structures, such as those from the Ediacaran *Cloudina*, show evidence of a nano-granular texture [71]. If the earliest examples of biomineralized structures already possess a nanometre-scale basic unit it is difficult to make a case for adaptation as there is no non-nano-granular texture to select against. Combining this observation of early structures with the likely independent acquisition of biomineralization among phyla [72,73], the origin of this nano-granular texture may also be explained by fabricational constraints related to the fundamental mechanisms of biologically controlled mineralization via particle attachment [74].

With these two examples in mind, we can return to a previous question: do the data presented here tell us anything about the evolution of the shell of *N. pompilius* or *A. argo*? Realistically, the answer is no. We do not delve into the evolutionary history of either species, nor do we discuss the ecology of neither animal nor the forces acting on the shell as a result of that ecology. Though remarkable, some of the properties of the shells discussed here, especially the frequency dependence of the argonaut shell, are not known to be functional.

## 4. Conclusion

The insights gained into the mechanics of not just the previously untested *Argonauta argo* shell but also the well tested nacreous shell of *Nautilus pompilius* demonstrate the importance of both multi-scale experiments and the incorporation of moisture control in testing biominerals. We uncover two very different approaches to achieve a structure that exhibits a combination of high deformability, stiffness, strength and toughness. In both cases, the performance of the shells on the scale of the entire animal is almost impossible to predict using local nanomechanical characterization methods. Furthermore, the energy dissipation mechanism of the argonaut was successfully demonstrated only by probing it under a habitat-like environment—in fully hydrated conditions. The results presented here emphasize the importance of scale and environment when attempting to understand the function of biological structures; how can we fully understand a structure while ignoring the environment, in which the structure evolved and performs? Moreover, how can we meaningfully hypothesize about function when removing the context of ecology?

Furthermore, we claim that although the studied organisms produce shells that provide them with sufficient mechanical support and protection against predation, the morphological, structural and crystallographic properties of the ultrastructures that comprise them are not necessarily the product of functional adaptation. In nacre, the nanoasperities can simply be the consequence of precursor nanoparticle accretion [75], mineral bridges—the result of epitaxial growth [76] and dove tailing—an outcome of space-filling requirements [77].

It is important to note that we are not trying to say that fabrication is the explanation for the commonality of this texture, nor are we saying that it is impossible for function to explain its origin either. Rather, if the case for adaptation is to be made it has to weighted against other potential explanations rather than assumed *a priori*. The tendency to explain all phenotypic traits of mineralized structures by adaptation, not as a hypothesis but as an obvious conclusion, does not provide meaningful insight into the actual evolution of these structures nor do such statements motivate further research into the potential morphogenetic constraints that might have actually been responsible in shaping their form. If future research wishes to address the question of functionality of the tissues being studied or their evolution, we should move away from the selectionist assumption that all observed features are, by default, products of adaptation.

## 5. Material and methods

### 5.1. Specimens and imaging

Two shells of *Nautilus pompilius* and one shell of *Argonauta argo* were used for this project. The shell of *Argonauta argo* was collected in 2012 during a mass stranding in Yoichi Bay. See [46] for further details. Both shells of *N. pompilius* were collected from the Philippines. Cryo-fractured samples were broken off from near the aperture of the shell, immersed in liquid nitrogen and manually fractured. Scanning electron microscopy images were made using an FEI Scios Dual Beam FIB/SEM. *A. argo* images were made using a voltage of 5 kV and a current of 50 pA. *N. pompilius* images were made using a voltage of 2 kV and a current of 25 pA. Samples of *A. argo* were embedded in poly(methyl methacrylate) and polished along their cross-section for EBSD analysis. EBSD was performed using an EDAX Hikari Plus EBSD system in low-vacuum conditions (0.2 mbar) at 1.6 nA and 15/20 kV.

### 5.2. Thermogravimetric analysis

The thermal stability of the samples was measured with a TGA system (SETARAM, SENSYS EVO TG-DSC). The shell samples were finely ground and the obtained powder was then used for measurement. At the outset, the sample was equilibrated at 25°C for 10 min to remove any absorbed moisture. After that, a heating ramp from 25 to 830°C was applied at a rate of 5°C min^−1^ under oxygen atmosphere to monitor the sequential decomposition of the sample contents.

### 5.3. Nanoindentation and nano-dynamic mechanical analysis

Indentation and nanoDMA experiments were performed on embedded and finely polished plane samples using a Hysitron/Bruker TI950 TriboIndenter equipped with xSol High-Temperature and Humidity Control Stage. A Berkovich diamond tip was used to measure the hardness and reduced modulus. The loading/unloading rate was set to 200 µN s^−1^, with a 5 s holding period at peak load of 1000 µN. The Oliver and Pharr approach [78] was used to analyse load–displacement curves in order to derive reduced modulus and hardness. A grid of indents was performed across a 96 × 36 µm^2^ and 1300 × 200 µm^2^ area, across the entire cross-sectional area of the shell wall of *A. argo* and *N. pompilius*, respectively. Indentation measurements were performed at four relative humidities: 30, 50, 70 and 90%. Additional nanoDMA measurements were performed on both shell sections at a relative humidity of 90% on two different regions of the shell: prismatic and nacre for *N. pompilius*, and mineral and organic rich areas for *A. argo*. The measurements were done with a set force of 800 µN and an oscillating force of 20 µN during a frequency sweep from 1 to 300 Hz.

### 5.4. Dynamic mechanical analysis

Rectangular bars were cut from each shell from the lateral area of the aperture. Several *N. pompilius* samples were also polished from the top and bottom to remove the inner and outer prismatic areas producing only a section of nacre. DMA was performed using an Anton Paar twin drive rheometer with an MCR502 rheometer drive and an MCR702 linear drive. Experiments were performed in three-point bending mode with a free length of 10 mm. Amplitude sweeps were performed for the three samples to compare the change in storage modulus values and displacements/applied forces relevant to the frequency sweep experiments to ensure the samples were within the linear viscoelastic range. All analyses were performed using a set force of 0.15 N and an oscillating force of 0.1 N. Frequency sweeps were performed between 0.1 and 10 Hz with 10 measurements per decade. ‘Dry’ measurements were made in air at ambient conditions (22°C, 60% RH). ‘Wet’ measurements were made in a full immersion cup after soaking each sample for a period of 8–14 h. Soaking time was determined after a series of tests on the *N. pompilius* shell as this shell is thicker than that of the argonaut and would require a longer rehydration time. First, dry measurements of the *N. pompilius* shell were taken. Second, the shell was soaked for 8–14 h and then measured in full immersion. These samples were allowed to air-dry and then measured again to ensure they returned to their pre-soaked parameters. Then the samples were soaked in water for two weeks and measured again. No significant difference was found when comparing data between the 8 and 14 h/two week rehydration times.

Each *N. pompilius* sample was measured four times and the results were averaged together with error bars calculated as 1 s.d. in the data. *A. argo* samples were also measured four times but this was divided between two orientations. Each *A. argo* sample was measured twice and then flipped over and measured twice again. This was done because the geometry of the argonaut shell is not flat and cannot be machined into a flat surface at the scale necessary for bending experiments.

## Data Availability

This article has no additional data.
